# HIV-1 reservoir inducibility and immune phenotypes after prolonged analytical treatment interruption: a long-term follow-up study

**DOI:** 10.1016/j.ebiom.2026.106411

**Published:** 2026-08-01

**Authors:** Cynthia Lungu, Tanvir Hossain, Raquel Crespo, Henrieke A.B. Prins, Kathryn S. Hensley, Casper Rokx, Shringar Rao, Jeroen J.A. van Kampen, David A.M.C. van de Vijver, Thibault Mesplède, Peter D. Katsikis, Rob A. Gruters, Yvonne M. Mueller, Tokameh Mahmoudi

**Affiliations:** a Department of Biochemistry, Erasmus University Medical Center, Rotterdam, Netherlands; b Department of Pathology, Erasmus University Medical Center, Rotterdam, Netherlands; c Department of Internal Medicine, Section Infectious Diseases, Erasmus University Medical Center, Rotterdam, Netherlands; d Department of Medical Microbiology and Infectious Diseases, Erasmus University Medical Center, Rotterdam, Netherlands; e Department of Viroscience, Erasmus University Medical Center, Rotterdam, Netherlands; f Department of Immunology, Erasmus University Medical Center, Rotterdam, Netherlands; g Department of Urology, Erasmus University Medical Center, Rotterdam, Netherlands

**Keywords:** Inducible HIV-1 reservoir, Tat/rev multiply spliced RNA, Immune phenotypes, CXCR3, Central memory CD4+ T cells, Trial endpoints, Analytical treatment interruption

## Abstract

**Background:**

Despite effective suppression of HIV-1 replication by antiretroviral therapy (ART), persistent viral reservoirs prevent cure and necessitate lifelong treatment. While short-term dynamics of HIV-1 rebound following treatment interruption have been extensively studied, the longer-term consequences of immune perturbation and transient viraemia for reservoir quality and immune architecture remain incompletely understood.

**Methods:**

We leveraged a rare historical human cohort that experienced prolonged viral rebound during a dendritic cell-based therapeutic vaccine study as a “perturbation model” to explore HIV-1 reservoir characteristics and immune remodelling more than a decade after ART resumption. We combined functional inducible HIV-1 reservoir measurements with high-dimensional immune phenotyping and included comparator analyses using a separate cohort of eight individuals with sustained ART-mediated suppression.

**Findings:**

We observed higher inducible HIV-1 reservoir activity in nine participants from the Dendritic Cell-Tat, Rev, and Nef (DC-TRN) trial who previously experienced viral rebound and subsequently achieved viral re-suppression on long-term ART (p = 0.01). This increase in inducible HIV-1 reservoir activity occurred in the absence of major changes in total or intact proviral DNA (p = 0.99, and p = 0.35, respectively). Immune profiling suggested persistent differences in memory T cell phenotypes, after long-term re-suppression on ART, including enrichment of CXCR3-expressing central memory CD4+ T cells that positively correlated with inducible *tat/rev* msRNA expression (r^2^ = 0.54, p = 0.03).

**Interpretation:**

Together, these findings suggest that immune perturbation and viral rebound may be associated with persistent changes in HIV-1 reservoir inducibility and immune architecture after viral re-suppression. Our study highlights a possible increase in inducible HIV-1 reservoir activity associated with a CXCR3+ memory T cell phenotype, supporting further evaluation of reservoir quality endpoints, candidate immunophenotypic correlates, and long-term monitoring strategies in contemporary remission trials.

**Funding:**

This research was supported by grants from Aidsfonds (P-60602, P-263, P-53302, P-53601, P-60804), MRACE (EMC2020-083); Horizon Europe (681032); Health Holland (LSHM19100-SGF and EMCLSH19023), and NWO (ZonMW-40-44600-98-333).

**Research in context**

**Evidence before this study**

We searched PubMed, Web of Science and Scopus for peer-reviewed publications from inception up to May 2026 using the terms “HIV” OR “HIV-1” AND “ATI” OR “analytical treatment interruption”. HIV-1 cure and remission strategies increasingly rely on controlled immune–viral perturbations (including antiretroviral treatment interruption) to evaluate intervention efficacy. Prior work has characterised short-term viral rebound dynamics and identified candidate transcriptional and immunologic signals that can precede or accompany rebound (e.g., viral transcripts and restriction factor signatures). However, the long-term consequences of rebound-associated perturbation on reservoir quality (inducibility and transcriptional activity, rather than bulk proviral DNA alone) and immune architecture remain incompletely understood, largely due to the scarcity of cohorts with deep longitudinal sampling many years after ART resumption.

**Added value of this study**

Using a rare historical cohort with prolonged viral rebound followed by long-term ART re-suppression, we provide a decade-long follow-up integrating functional reservoir assays and high-dimensional immunophenotyping. Our findings suggest increased inducible HIV-1 reservoir activity despite broadly stable measures of total/intact proviral DNA, supporting the concept that reservoir quality can diverge from reservoir quantity. We also observed persistent differences in memory T-cell phenotypes and an association between CXCR3-expressing central memory CD4+ T cells and inducible *tat/rev* msRNA expression. These findings highlight a candidate phenotypic axis that may link immune trafficking programs to inducible HIV-1 reservoir activity.

**Implications of all the available evidence**

Together, available evidence and our findings support further evaluation of cure trials beyond plasma viral load and DNA-based reservoir quantification, including reservoir quality endpoints and scalable immunophenotypic biomarkers. The observed persistence of inducible HIV-1 reservoir activity and CXCR3-linked immune signatures long after viral re-suppression suggests that longer-term monitoring may provide additional insight into immune and reservoir remodelling after ATI. Larger prospective studies will be needed to determine whether these measures can inform risk stratification or monitoring strategies in contemporary remission trials, including settings where analytical treatment interruption is minimised or avoided but immune–viral perturbations may still occur.

## Introduction

More than four decades after the discovery of human immunodeficiency virus type 1 (HIV-1), a curative intervention remains elusive. Combination antiretroviral therapy (ART) effectively suppresses viral replication and has transformed HIV-1 infection into a manageable chronic condition, yet it does not eradicate the virus. A heterogenous reservoir of latently infected cells^1^, ^2^, ^3^ can persist for decades despite potent ART and reignite systemic viraemia upon ART interruption or failure.^4^^,^^5^ These reservoirs are exceedingly rare—estimated at 1 to 1000 infected cells per million CD4+ T cells—and consist of a mixture of replication-competent and defective proviruses that current assays cannot reliably distinguish.^6^, ^7^, ^8^ Furthermore, tissue-based reservoirs, within lymphoid organs, the gastrointestinal tract, and the central nervous system further complicate eradication efforts due to limited accessibility and distinct immunological microenvironments.^9^^,^^10^

Understanding the qualitative features of the HIV-1 reservoir that govern persistence and reactivation remains a central challenge in cure research. While analytical treatment interruption (ATI) has been widely used to functionally assess curative strategies,^11^, ^12^, ^13^ most studies have focused on short-term virologic and immunologic outcomes during or immediately after viral rebound.^14^, ^15^, ^16^, ^17^, ^18^, ^19^ These studies have provided important insights into rebound kinetics and immune control but offer limited information on whether immune perturbation and viral replication leave durable biological imprints once ART-mediated suppression is re-established.

Importantly, the HIV-1 reservoir is established very early during infection and can rapidly expand during periods of uncontrolled viraemia.^20^, ^21^, ^22^ Plasma HIV-1 RNA levels typically rebound within weeks of ART interruption, yet the extent to which the duration and magnitude of viraemia—or the immune perturbations accompanying it—drive long-term changes in reservoir composition and immune architecture remains incompletely understood. While modern ATI protocols have been refined to minimise clinical risk through close monitoring and early ART resumption,^23^, ^24^, ^25^, ^26^, ^27^ these designs, with brief follow-up, inherently limit the ability to study long-term biological consequences of viral rebound and immune activation following re-suppression. Historical cohorts exposed to prolonged interruptions therefore represent a unique and irreplaceable opportunity to interrogate the durability of HIV-1 reservoirs and immune perturbations that cannot be readily examined in contemporary trials. Emerging evidence suggests that ATI may induce subtle but lasting effects on reservoir composition and immune phenotypes even after viral re-suppression,^16^^,^^28^ yet comprehensive longitudinal data extending beyond several years are scarce.

In this study, we leveraged a rare cohort of individuals who participated in a phase I/IIa clinical trial evaluating autologous Dendritic cell (DC)-Tat, Rev and Nef Immunotherapy for people living with HIV (DC-TRN) that included ATI nearly two decades ago.^29^ These individuals were re-enrolled more than ten years after ART resumption and sustained viral suppression, providing an unprecedented window into the long-term immunovirological consequences of immune perturbation and viral rebound. Prior work in this cohort demonstrated profound and durable transcriptional changes following vaccination and ATI.^30^ Building on this foundation, we applied an integrated approach combining HIV-1 proviral reservoir quantification, inducibility profiling, and high-dimensional immune phenotyping to characterise long-term alterations in reservoir quality and immune architecture.

## Methods

### Ethics statement

Participants of the DC-TRN trial provided written informed consent prior to inclusion in accordance with the regulations of the institutional Medical Ethics Committee at Erasmus Medical Center, Rotterdam (MEC-2005-227). The trial was conducted in full conformity with the principals expressed in the Declaration of Helsinki and participants provided new written consent for the long-term follow-up sampling and analysis in 2021 (MEC-2012-583). The remaining material from the DC-TRN trial was stored in a registered biobank, which was approved by the Erasmus Medical Center Medical Ethics Committee (MEC-2022-0060). The HIV biobank is described at https://eheg.nl/en/Our-Projects/HIV-Biobank. Samples used as controls were obtained from participants of studies at Erasmus Medical Center: Ex Vivo study, HIV Biomarker study and LUNA clinical trial, with approvals from the institutional Medical Ethics Committee at Erasmus Medical Center (MEC-2012-583, MEC-2016-148, and MEC-2017-476, respectively). All control participants provided written informed consent for sample collection, storage, and use in research in accordance with the relevant study protocols and ethics approvals.

### Participant selection criteria and bias mitigation

The DC-TRN trial was conducted across two country sites: the Netherlands and Belgium. To reduce potential bias arising from site-related differences, sample availability, and laboratory processing, the present analysis was restricted to ATI participants from the Netherlands site for whom archived samples were available and suitable for the planned analysis and for whom additional leukapheresis sampling was feasible. The parent DC-TRN trial enrolled male participants only; consequently, all ATI participants included in the present analysis were male. Because the DC-TRN trial did not include a placebo control arm, comparator participants were selected from ongoing Erasmus MC cohort studies in the Netherlands. These participants had HIV-1 subtype B infection, no history of ATI and were matched as closely as possible to the ATI participants on key clinical parameters, including sex, age, CD4+ T cell nadir, CD4+ T cell count at sampling, and duration of ART (Supplementary Table S1).

### Plasma HIV-1 RNA monitoring

Quantification of plasma HIV-1 RNA was performed on three different platforms available at various longitudinal timepoints during DC-immunotherapy, at treatment interruption and on ART. Between 2002 and 2008, quantification was performed using the Roche Amplicor HIV-1 RNA monitor test (version 1.5) (LOQ 50 copies/mL). After 2009 plasma HIV-1 RNA was quantified using the COBAS TaqMan Assay (Roche); LOQ 20 copies/mL. As of 2021, plasma HIV-1 RNA was quantified using the Aptima HIV-1 Quant Dx assay; LOQ 30 copies/mL.

### Clinical sample collection and cell isolations

Cryopreserved PBMCs obtained from large blood draws or leukapheresis material collected from the study participants during the trial phase and post ATI were used for this study. Upon thawing PBMCs, CD4+ T cells were isolated by negative magnetic selection using the EasySep Human CD4+ T cell Enrichment kit (StemCell Technologies) according to the manufacturer's instructions. The isolated CD4+ T cells were either directly used to quantitate HIV-1 DNA/RNA levels or treated with 100 ng/mL phorbol 12-myristate 13-acetate (PMA) and 1 μg/mL ionomycin to enhance viral reactivation.

### Intact proviral DNA assay (IPDA)

The levels of intact proviral DNA were assessed using lysed extracts of CD4+ T cells. Nucleic acid was isolated from at least 2 × 10^6^ CD4+ T cells using the PCI extraction method. Digital PCR (dPCR) was performed on an Absolute Q digital PCR platform (Thermo Fisher Scientific) using primer and probe sets targeting the packaging signal (Ψ) and Envelope gene (*env*), following the manufacturer's instructions and adapted intact proviral DNA assay protocol.^7^^,^^31^^,^^32^ The duplex HIV-1 dPCR reaction mix consisted of 1.8 μL Absolute Q DNA Digital PCR Master Mix 5X (Thermo Fisher Scientific), 1 μL packaging signal (Ψ) target primer/FAM-probe mix (10x), 1 μL *env* target primer/HEX-probe mix (10x), 700 ng gDNA, diluted in nuclease-free H2O to a final reaction volume of 9 μL. The dark competition probe for APOBEC3G hypermutations in the *env* region was omitted from this reaction setup. Measurements of the cellular gene ribonuclease P/MRP subunit p30 (RPP30) in a replicate well were used to calculate the frequency of CD4+ T cells. The duplex RPP30 dPCR was set up similarly using 7 ng of gDNA, as previously described, to calculate the DNA shearing index and measure input cell number. The following dPCR programme was used: Enzyme activation at 95 °C for 10 min; Denaturation at 95 °C for 30s; Annealing/Extension at 60 °C for 1 min (45 cycles), followed by enzyme deactivation at 98 °C for 10 min. Data analysis was performed using the QuantStudio Absolute Q Software Version 6.3. The primer and probe sequences used are provided in Supplementary Table S2.

### Fluorescence In Situ Hybridisation-flow cytometry (FISH-flow)

The frequency of cells expressing gag-pol viral RNA (vRNA) and p24 protein were assessed using the Primeflow RNA Assay (Thermo Fisher Scientific) following the manufacturer's instructions and as described previously.^33^^,^^34^ Briefly, CD4+ T cells were isolated from people with HIV-1 donor PBMCs by negative magnetic selection using the EasySep Human CD4+ T cell Enrichment kit (StemCell Technologies) according to the manufacturer's instructions. A minimum of 20 × 10^6^ CD4+ T, resuspended at 1.5 × 10^6^ cells/mL in RPMI-1640 medium supplemented with 10% FBS, 100 μg/mL penicillin-streptomycin and Raltegravir (3 μM) and rested for at least 4 h in a humidified incubator at 37 °C and 5% CO_2_. Cells were then treated with 100 ng/mL PMA and 1 μg/mL ionomycin to enhance viral reactivation. Eighteen hours post stimulation, cells were washed, pellets resuspended in PBS, and counted using an automated cell counter (Countess II, Thermo Fisher Scientific). At least 10 × 10^6^ cells, with >55% viability, were subjected to the FISH-flow assay. CD4+ T cells were first stained in Fixable Viability dye 780 (Thermo Fisher Scientific) for 20 min at room temperature (1:1000 dPBS) and then fixed and permeabilized using reagents from the Primeflow RNA assay kit according to manufacturer's instructions. After permeabilization, the cells were stained with two p24 antibodies; anti-p24 KC57-FITC (Beckman Coulter, 6604667) and anti-p24 28B7-APC (MediMabs, MM-0289-APC), incubated in the dark for 30 min at room temperature, and an additional 30 min at 4 °C. Cells were then washed and resuspended in RNA wash buffer with RNAsin. mRNA was labelled with a set of 40 probe pairs against the GagPol region of vRNA (catalogue number: GagPol HIV-1VF10-10884, Thermo Fisher Scientific) diluted 1:5 in the probe diluent provided in the kit. Target mRNA hybridisation was carried out for 2 h at 40 °C. Samples were washed to remove excess probes and stored overnight, at 4 °C, in the presence of RNAsin. Signal amplification was then performed by sequential 1.5-h, 40 °C incubations with the pre-amplification and amplification mix. Amplified mRNA was labelled with fluorescently tagged probes for 1 h at 40 °C. Samples were washed, resuspended in 200–300 μL storage buffer and up to 5 × 10^6^ cells were acquired on a BD LSR Fortessa Analyser. Gates were set using stimulated, uninfected CD4+ T cells. Data were analysed using the FlowJo V10 Software (Treestar).

### Specific Quantitation of inducible HIV-1 reservoir by LAMP (SQuHIVLa)

The frequency of cells spontaneously and inducibly expressing *tat/rev* multiply spliced (ms) HIV-1 RNA was assessed using a SQuHIVLa assay according to the protocol described elsewhere.^35^ Briefly, 2–5 × 10^6^ total CD4+T cells were resuspended (1.5 × 10^6^ cells/mL) in culture RPMI-1640 medium supplemented with 10% FBS and 100 μg/mL penicillin-streptomycin, and rested for 6 h in a humidified incubator at 37 °C and 5% CO_2_. After incubation, some cells were left untreated and directly used to determine basal viral RNA expression or treated with 100 ng/mL PMA and 1 μg/mL ionomycin for 12 h to induce viral reactivation. Cells from either condition were washed twice in RPMI 1640 media with 3% FBS, then resuspended in PBS and counted using an automated cell counter (Countess II, Thermo Fisher Scientific). A minimum of 0.5 × 10^6^ CD4+ T cells, with >50% viability, after PMA/ionomycin treatment were analysed per SQuHIVLa assay performed on a CFX96 Touch Real-Time PCR Detection System thermocycler (BioRad) as described elsewhere.^35^

### One-step *tat/rev* induced limiting dilution assay (TILDA)

The frequency of cells expressing *tat/rev* multiply spliced (ms) HIV-1 RNA was assessed using a modified TILDA protocol^36^ i.e., by direct amplification of *tat/rev* msRNA using a one-step RT-qPCR approach. Briefly, 2–5 × 10^6^ CD4+ T cells isolated from people with HIV donor PBMCs were rested and treated with PMA/ionomycin as described for the other inducible reservoir assays. A minimum of 0.5 × 10^6^ CD4+ T cells, with >50% viability, after treatment with PMA/ionomycin were analysed per assay. CD4+ T cells were added in limiting dilution, ranging from 3 × 10^4^ cells - 3 × 10^3^ cells (22–24 replicates), to a white 96-well qPCR plate (Biorad) containing 20 μL One-step RT-qPCR reagent mix; 5 μL Luna Probe One-Step RT-qPCR 4X Mix with UDG (No ROX) (New England BioLabs), 0.1 μL *tat* 1.4 forward primer and 0.1 μL *rev* reverse primer (both at 100 μM), 0.8 μL HIV *tat/rev* probe (5 μM), and 14 μL nuclease free water to a final reaction volume of 25 μL. The primer and probe sequences used, as published.^36^ The qPCR plates were sealed, briefly centrifuged at 600 × *g* for 1 min, and a CFX96 Touch Real-time PCR instrument (Biorad) was used to run the following thermocycling program: carry over prevention step at 25 °C for 30s, reverse transcription at 55 °C for 10 min, initial denaturation at 95 °C for 3 min followed by 50 cycles of denaturation at 95 °C for 10s and extension at 60 °C for 1 min. The amplification curves in all positive wells at each dilution manually inspected after each run and the number of positive wells were used to determine the frequency of cells positive for *tat/rev* msRNA using the maximum likelihood method.

### In-depth immunophenotyping of the major cell subsets present in human peripheral blood

PBMCs from post-ATI participants (at T6) and historical HIV+ controls, matched by age, sex and time on ART (Supplementary Table S1), were thawed and stained with a 45-colour flow cytometry panel and data was read on a 5-laser Aurora spectral flow cytometer (Cytek Biosciences, CA). Staining of the samples was performed following the OMIP-109 platform with minor modifications.^37^ Specifically, we replaced the live/dead stain for anti-Annexin V conjugated antibody (catalogue number: A23202, Thermo Fisher Scientific) to exclude both dead and apoptotic cell populations and added 2.5 mM CaCl_2_ to all buffers. In addition, to better define a CD20+CD19+ population, we added anti-CD20 antibody as a last step of the sequential staining for 5 min at room temperature, before staining with the full remaining antibody cocktail. Spectral unmixing and compensation check was performed in SpectroFlo (version 2.2.0). Flow cytometry data clean-up was performed using the flowAI algorithm embedded in the OMIQ data analysis software (www.omiq.ai). Our downstream data processing workflow included scaling adjustments, sub-sampling (max equal) and manual gating to define live populations of single cells. For high-dimensional analysis, we used SPADE^38^ algorithm for clustering and UMAP^39^ projections for dimensionality reduction and 2D visualisation of defined clusters. For analysis of cluster abundance differences between groups, we used edgeR algorithm (FDR <0.05) and volcano plots for visualisation. For multivariate statistical analysis of the median fluorescent expression of activation and exhaustion markers in specific clusters, we used Significant Analysis of Microarrays (SAM) plot algorithm with FDR <0.01. For manual gating of cell populations, the OMIP-109 gating strategy was followed.^37^ All programs used for analysis are embedded within the OMIQ data analysis software. RRID identifiers for the antibodies used, where available, are provided in Supplementary Table S3.

### Sample size and statistical analysis

The study size was determined by the number of eligible DC-TRN participants from the Netherlands site for whom archived samples were available and suitable for the planned analyses and for whom additional leukapheresis sampling was feasible. Participants from the Belgian study site were not included because archived samples required for the planned analyses were not available. No formal sample size calculation was performed because this was a secondary exploratory analysis nested within the DC-TRN study. Consequently, the analyses were considered hypothesis-generating and were not powered to detect small between-group differences.

Data are presented as median with Interquartile range (IQR). Spearman rank correlation coefficients were calculated for non-normally distributed variables. All statistical tests were two-tailed and P-values <0.05 was considered statistically significant.

Reported limits of detection for HIV FISH-flow assays vary by platform; for the HIV RNA/Gag FISH-flow assay, the detection limit has been reported as approximately 0.5–1 *gagpol* mRNA+/Gag protein+ cell per million CD4 T cells.^40^ Inducible reservoir quantification assays such as SQuHIVLa and TILDA are based on maximum-likelihood statistics, with limits of detection dependent on the initial cell input. Measurements below the limit of detection were assigned an arbitrary value of 0.5 cells per million for visualisation purposes only and were excluded from statistical analyses. One outlier from the T6 SQuHIVLa dataset was identified by robust regression and outlier removal (ROUT; Q = 10%) and excluded from subsequent statistical analyses. Mixed effects model analyses, Tukey's multiple comparison tests, and Dunn's multiple comparison tests were used to compare viral reservoir frequencies across timepoints. Adjusted P-values were calculated and P < 0.05 was considered statistically significant. Biological replicates (i.e., samples at longitudinal timepoints) were included in most experiments except for inducible HIV reservoir quantification, owing to limited availability or quality of clinical material (particularly baseline samples). Technical replicates were performed as follows: for intact proviral DNA quantification by dPCR, at least two technical replicates; for FISH-flow, experiments were not technically replicated given the limited clinical material; for SQuHIVLa and 1-step TILDA, at least two independent experiments were performed where sufficient clinical material was available. Graphs and statistical analyses were generated using GraphPad Prism 10 for MacOS, Version 10.5.0 (673).

### Role of funders

The funders had no role in study design, data collection, analysis, interpretation of the results or the decision to publish the manuscript.

## Results

### Longitudinal clinical outcomes following prolonged ATI and resumption of ART

**Fig. 1:**
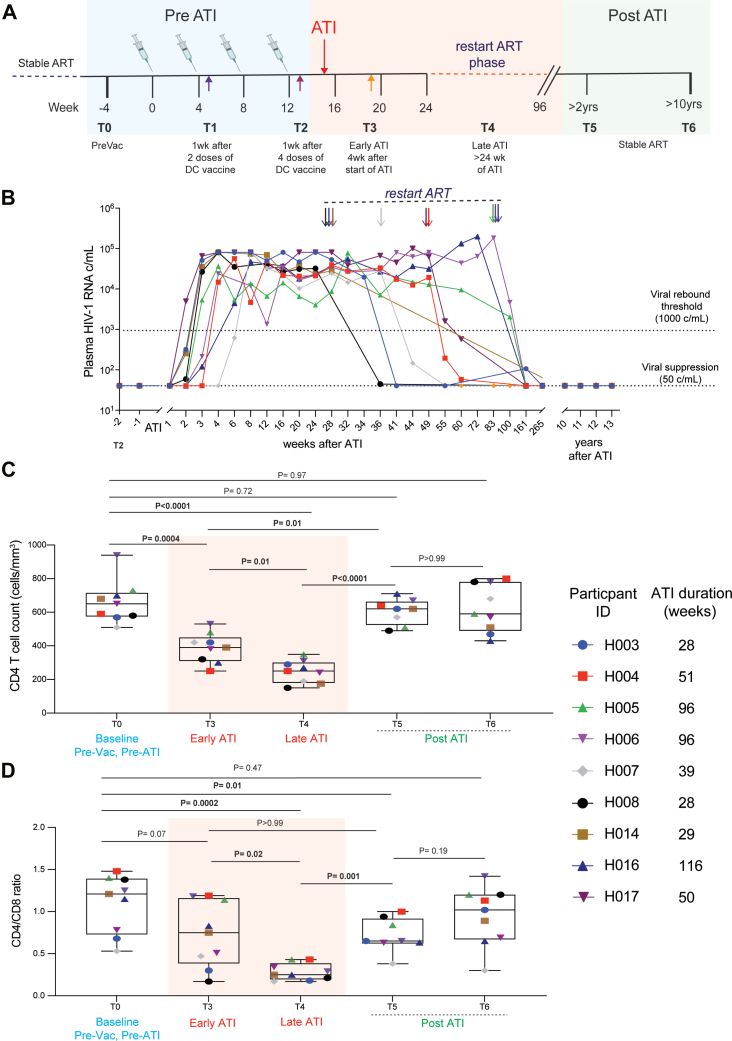
**Longitudinal Plasma HIV-1 RNA and T cell dynamics. A**) Schematic overview of the longitudinal study phase during the DCTRN vaccination trial, ATI (pre-Vac to 96 weeks) and after 13 years on suppressive ART. The study timepoints are indicated as T0 to T6. The start of analytical treatment interruption (ATI) is shown with a red downward arrow. The period during which nine participants restarted ART is indicated using a dotted horizontal line. Participants received standard care after restarting ART. Timepoint 5 (T5) was a routine care visit with standard blood draw, and no extra samples were collected for reservoir analyses. Timepoint 6 (T6) was an extra visit with large blood collection at a median of 12.4 years after resuming ART. **B)** Longitudinal plasma HIV-1 RNA kinetics after ATI. The downward coloured arrows mark the time of ART resumption. **C**) CD4+ T cell counts and **D**) CD4/CD8 ratio dynamics measured at selected study timepoints. Box plots with mean and range are shown. The shaded bars depict the ART interruption phase. Statistics inferred using a mixed effect analysis with multiple comparisons of the means (Tukey–Kramer test). Adjusted P values are shown above the graphs.

Our study cohort offers a rare opportunity to examine long-term immunovirological outcomes following vaccination and analytical treatment interruption (ATI). Seventeen chronically treated individuals with HIV-1 subtype B underwent prolonged ATI during a non-randomised, phase I/IIa autologous dendritic cell (DC)-based immunotherapy trial starting in 2006 (International Clinical Trial Registry Platform: Netherlands Trial Registry No. NTR2198).^29^ At the time of enrolment for the clinical trial, all 17 participants had been on suppressive ART for a median of 4.8 years (interquartile range (IQR): 4.6–5), with undetectable plasma HIV-1 RNA levels (<50 copies/mL) and CD4+ T cell counts consistently above 500 cells/μL.^29^ While the DC vaccine was shown to be safe and well-tolerated, the elicited immune responses were not sufficient to control viral replication in absence of ART. All participants experienced viral rebound, with plasma HIV-1 RNA levels exceeding 1000 copies/mL within two to eight weeks after ATI.^29^ The median time off ART was 50 weeks (IQR: 28.5–96), after which therapy was resumed according to protocol-defined CD4+ T cell thresholds or earlier treatment guidelines.^29^ Detailed clinical characteristics and a treatment history of the DC-TRN participants before and after ATI are described elsewhere.^29^ In 2021, more than a decade later, we re-enrolled nine of the DC-TRN trial participants, who were in care at Erasmus Medical Center in the Netherlands, for follow-up sampling to enable a unique longitudinal assessment spanning pre- and post-vaccination, ATI phase, and long-term ART re-suppression. The clinical characteristics of the nine participants included in the present study are summarised in Table 1. Since the DC-TRN trial did not have a control group, a separate cohort of individuals with sustained ART-mediated suppression (n = 8) was included for comparator analyses (Supplementary Table S1). Blood samples were collected at six defined time points: baseline (T0, prior to DC vaccination); pre-ATI (T1, one week after the second DC vaccination; and T2, one week after the fourth dose); early ATI (T3, four weeks after ATI initiation); late ATI (T4, >24 weeks into ATI and immediately prior to ART resumption)^29^ and post-ATI (T5, >2 years after ART resumption and T6, >10 years after ART resumption) (Fig. 1A). Based on retrospective clinical data, plasma antiretroviral drug levels measured one month after resuming ART were within therapeutic ranges. Plasma viraemia re-suppressed to below 50 copies/mL in all individuals within 18 months of ART resumption (Fig. 1B). Over the following decade, three participants experienced isolated viral blips (50–400 copies/mL) (see note under Table 1), with no evidence of ART failure, non-adherence, or use of potential HIV-1 reservoir-targeting therapies. A significant decline in CD4+ T cell counts during ATI was observed, particularly at the late ATI time point (T4), where median counts dropped markedly compared to baseline (P < 0.0001) (Fig. 1C). CD4+ T cell counts gradually recovered after ART resumption, reaching levels comparable to T0 by T6 (>10 years post-ATI; P = 0.97). Similarly, the CD4+/CD8+ T cell ratio decreased during ATI, with a significant reduction at T4 (vs. T0, P = 0.0002) (Fig. 1D). Although CD4+ T cell counts had recovered to levels comparable to T0 by T5, the CD4+/CD8+ T ratio remained significantly lower than baseline at two years after ART resumption (T5 vs. T0, P = 0.01), and normalised only by T6 (P = 0.47), suggesting more prolonged perturbation of the CD8+ T cell compartment after ART resumption (Fig.1D). These data indicate that recovery of absolute CD4+ T cell counts preceded normalisation of the CD4+/CD8+ T cell ratio after prolonged ATI.

**Table 1:** Longitudinal clinical characteristics of DC-TRN trial participants (n = 9).

	Median	IQR	Range
Sex assigned at birth	Male (100%)		
Age at DC vaccination (T0)	45	41.5–53	37–56
Age on stable ART post intervention (T6)	60	56.5–68	52–71
Year of HIV diagnosis	1997	1994–1999	1988–2000
Time between HIV diagnosis and ART initiation (weeks)	32	18.5–198.0	2–442
Time on ART before vaccination (years)	9.9	7.0–10.85	3.7–11.9
Time on ART with undetectable plasma HIV RNA before DC vaccination (years)	4.8	4.6–5.0	2.8–5.3
Peak plasma HIV RNA pre-ART (log10 c/mL)	5.01	4.86–5.12	4.73–5.31
Nadir CD4+ T cell count	230	155–5.12	70–290
CD4+ T cell count pre-ART initiation	370	325–435	300–650
CD8+ T cell count pre-ART initiation	1850	1360–2370	1120–2890
CD4/CD8 ratio pre-ART initiation	0.24	0.14–0.33	0.12–0.38
CD4+ T cell count before DC vaccination (T0)	650	575–715	510–940
CD8+ T cell count before DC vaccination (T0)	610	470–835	400–970
CD4/CD8 ratio baseline (T0)	1.21	0.73–1.39	0.53–1.48
**Analytical ART interruption**			
Time to viral rebound >50 c/mL (weeks)	3.0	2.3–3.65	2.0–6.3
Time to viral rebound >500 c/mL (weeks)	3.3	2.65–5.15	2.0–6.3
Time to viral rebound >1000 c/mL (weeks)	3.3	2.65–5.15	2.0–8.3
Time to viral rebound >50,000 c/mL (weeks)	6.3	3.15–20.15	2.0–40.0
Lowest CD4+ T cell count	250	175–285	150–290
Peak CD8+ T cell count	1370	1195–2040	1060–4400
Last CD4+ T cell count before ART resumption (T4)	250	180–300	150–350
Last CD8+ T cell count before ART resumption (T4)	820	685–1090	580–1650
CD4/CD8 ratio before ART resumption (T4)	0.25	0.20–0.39	0.17–0.43
Weeks off ART	50	28.5–96.0	28–116
**ART resumption**			
Time to viral resuppression <50 c/mL (months)	8.5	4.0–16.0	3.0–18.0
Time on ART post resumption, early (years); T5	2.3	2.2–3.2	2.0–3.2
CD4+ T cell count (T5)	620	525–663	490–710
CD8+ T cell count (T5)	975	617.5–1098	520–1510
CD4/CD8 ratio (T5)	0.65	0.62–0.92	0.38–1.0
Time on ART post resumption, late (years); T6	12.4	11.9–13.05	11. - 13.3
Time on ART with undetectable plasma HIV RNA (years); T6	10.5	7.8–11.5	6.2–12.9
CD4+ T cell count (T6)	590	490–780	430–800
CD8+ T cell count (T6)	650	520–770	460–2240
CD4/CD8 ratio (T6)	1.02	0.67–1.20	0.30–1.42

T0: baseline, before DC vaccination, T4: Late ATI.

T cell counts: cells/mm^3^; c/mL: copies/mL; IQR: Interquartile range.

Three participants with viral blips (50–400 c/mL) since viral re-suppression. **H003**—255 c/mL (Oct 2009), 132 c/mL (April 2010), 59 c/mL (Jan 2014); **H006** (slow to suppress after restarting ART)—57 c/mL and 228 c/mL (Nov 2013); **H008** (slow to suppress after restarting ART); **H017**—71 c/mL (Jan 2016).

### Molecular dissection of the HIV-1 reservoir reveals long-term persistence and increase of inducible latent reservoir Post-ATI

Building upon our longitudinal clinical analyses, we next performed in-depth molecular reservoir quantification to evaluate changes in HIV-1 persistence markers. The extended follow-up and availability of samples at all key phases—pre-ATI, during ATI, and more than a decade after ART resumption—allowed us to comprehensively profile the HIV-1 reservoir across three distinct molecular levels: proviral DNA, viral RNA transcription, and Gag p24 protein translation. This multi-layered assessment provides unique insight into how clinical interventions, including ATI, may influence reservoir composition and stability, helping refine strategies to evaluate the impact of future ATI-based cure interventions.

**Fig. 2:**
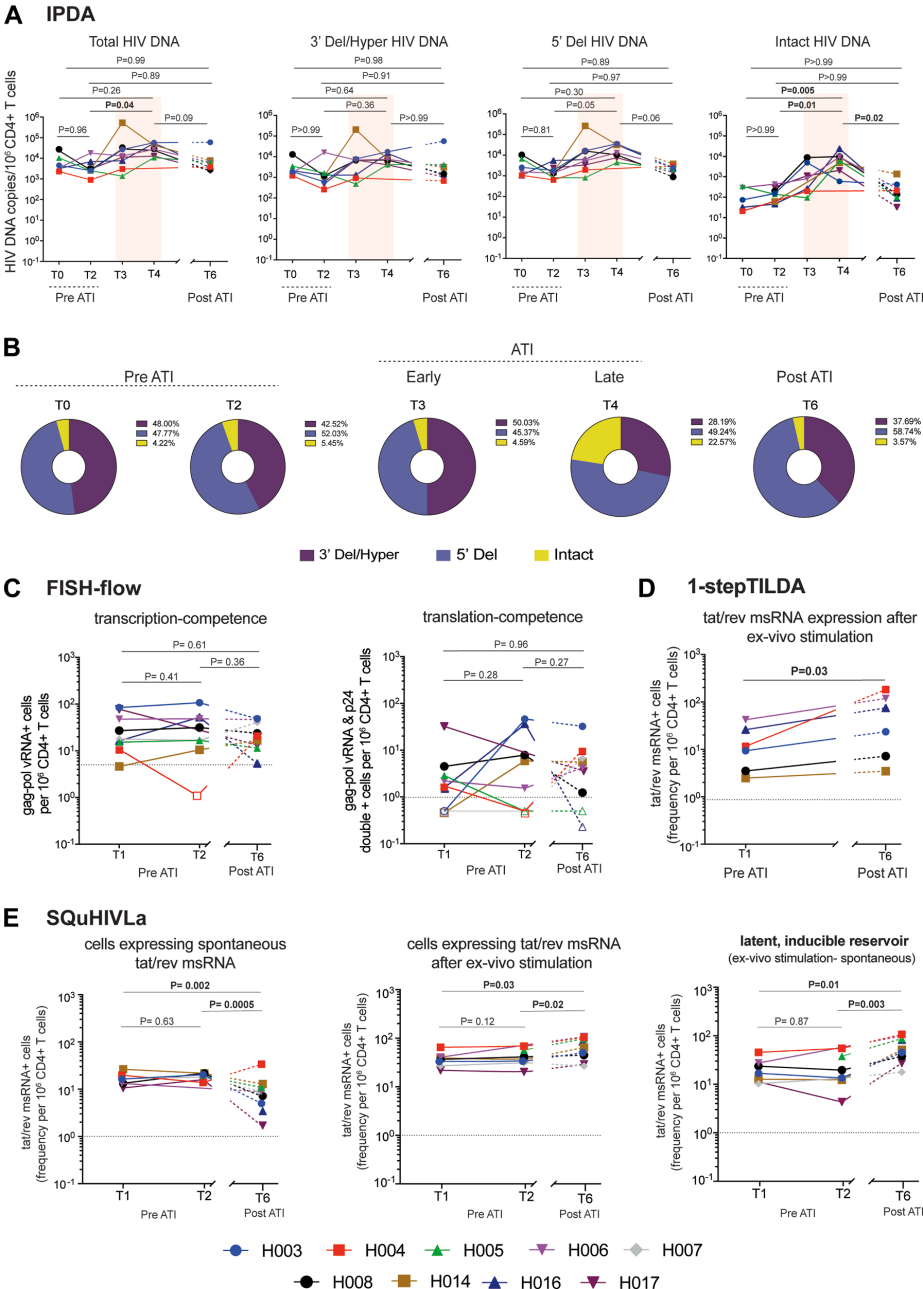
**HIV-1 reservoir dynamics after ATI and long-term ART-mediated viral re-suppression. A)** Changes in levels of total, intact and defective proviral DNA in peripheral CD4+ T cells in response to DC vaccination, ATI and post-ATI (n = 8). Mixed effects model analyses, Tukey's multiple and Dunn's multiple comparison tests were used to compare viral reservoir quantities across timepoints (Adjusted P values are shown). The shaded bars depict the ATI phase. **B**) Change in proportions of intact to defective HIV-1 DNA. **C**) Change in CD4+ T cells expressing unspliced *gag-pol* viral RNA and cells co-expressing *gag-pol* vRNA and Gag p24 protein after ex vivo treatment with PMA/ionomycin for 18 h, measured using FISH-flow (n = 9). Open symbols depict quantities below the assay limit of detection (dotted line) The means of 2–3 measurements were used in subsequent statistical analyses. **D**) Change in CD4+ T cells expressing *tat/rev* msRNA after ex vivo treatment with PMA/ionomycin for 12 h, measured using 1-step TILDA (n = 6). The mean of 2 measurements was used in subsequent statistical analyses. **E**) Change in CD4+ T cells spontaneously expressing *tat/rev* msRNA (*in vivo* response) and inducibly expressing *tat/rev* msRNA after ex vivo treatment with PMA/ionomycin for 12 h, measured using SQuHIVLa (n = 9). Mixed effects model analyses and Tukey's multiple comparison test were used to compare viral reservoir quantities across timepoints. The means of 2–3 measurements were used in subsequent statistical analyses. Adjusted P values are shown.

We first quantified total, defective (5′ or 3′ deletion), and intact HIV-1 proviral DNA using the Intact Proviral DNA Assay (IPDA) across all key timepoints (Fig. 2A).^7^ DC vaccination alone did not alter total HIV DNA levels between T0 and T2 (P = 0.96). However, the proportions of intact proviral DNA increased in three participants, and decreased in three participants (Supplementary Fig. S1). During ATI, the levels of intact proviral DNA increased at both early (T3) and late (T4) ATI, coinciding with active viraemia (Fig. 2A and B). Notably, by T6 (>10 years post-ATI), levels of total, defective, and intact proviral DNA were overall similar to those measured at T0 and T2, although four participants showed a notable upward trend in intact proviral DNA compared to T2 (Fig. 2A and B, Supplementary Fig. S1).

While measurement of the intact viral reservoir at the DNA level by IPDA is considered a marker of replication-competence, the integration site of intact proviruses directly affects inducibility and transcriptional output.^41^, ^42^, ^43^ To specifically profile proviral transcriptional activity, we next assessed viral RNA expression using FISH-flow, which detects CD4+ T cells expressing unspliced *gag-pol* HIV-1 RNA following ex vivo PMA-ionomycin stimulation.^33^ Median frequencies of *gag-pol* vRNA+ cells remained stable across T1 (17.85 per 10^6^ CD4+ T cells; IQR: 12.84–62.60), T2 (24.11; IQR: 12.05–51.34), and T6 (17.60; IQR: 12.32–43.62), indicating persistence of the transcription-competent viral reservoir over time (Fig. 2C). Parallel analysis of the translation-competent reservoir, cells co-expressing *gag-pol* vRNA and Gag p24 protein, revealed consistently low to undetectable frequencies across all timepoints, consistent with previous literature and supporting the notion that viral translation is tightly restricted under suppressive ART (Fig. 2C).^33^^,^^44^

Although HIV-1 RNA expression is widely used as a surrogate for “latency reversal” in cells from participants following ex vivo treatment or after administration of latency reversing agents *in vivo*,^45^ most HIV-1 RNA expression comes from defective proviruses. The majority, over 94% of proviruses, is 3′ defective and expresses 3’ defective RNA.^46^ *Tat/rev* spliced RNA transcripts are less likely to arise from defective proviruses, and are thus considered a more accurate surrogate marker of replication-competence. Therefore, to more specifically quantify the inducible latent HIV-1 reservoir, we applied two complementary assays targeting *tat/rev* multiply spliced RNA (msRNA): SQuHIVLa^35^ and 1-step TILDA. At T1 and T2, spontaneous *tat/rev* msRNA expression was comparable between both timepoints (medians: 16.32 and 19.31 per 10^6^ CD4+ T cells, respectively) but declined significantly by T6 (median 8.01; IQR: 3.84–10.38), with statistically significant differences between T6 vs. T1 (P = 0.002) and T6 vs. T2 (P = 0.001), suggesting reduced basal transcriptional activity post-ATI (Fig. 2E). However, upon ex vivo stimulation with PMA-ionomycin, the frequencies of *tat/rev* msRNA+ cells were significantly higher at T6 than at both T1 and T2 (P = 0.03 and P = 0.02, respectively), indicating increased inducibility of latent proviruses (Fig. 2E). These findings were corroborated by 1-step TILDA, which showed similar trends (P = 0.03) (Fig. 2D). Calculation of the latent, inducible reservoir, defined as the difference between induced and spontaneous *tat/rev* msRNA+ cells, revealed a statistically significant increase at T6 compared to T1 and T2 (P = 0.01 and P = 0.003, respectively) (Fig. 2E), suggesting an increase of inducible but transcriptionally silent proviruses after more than a decade after ATI.

**Fig. 3:**
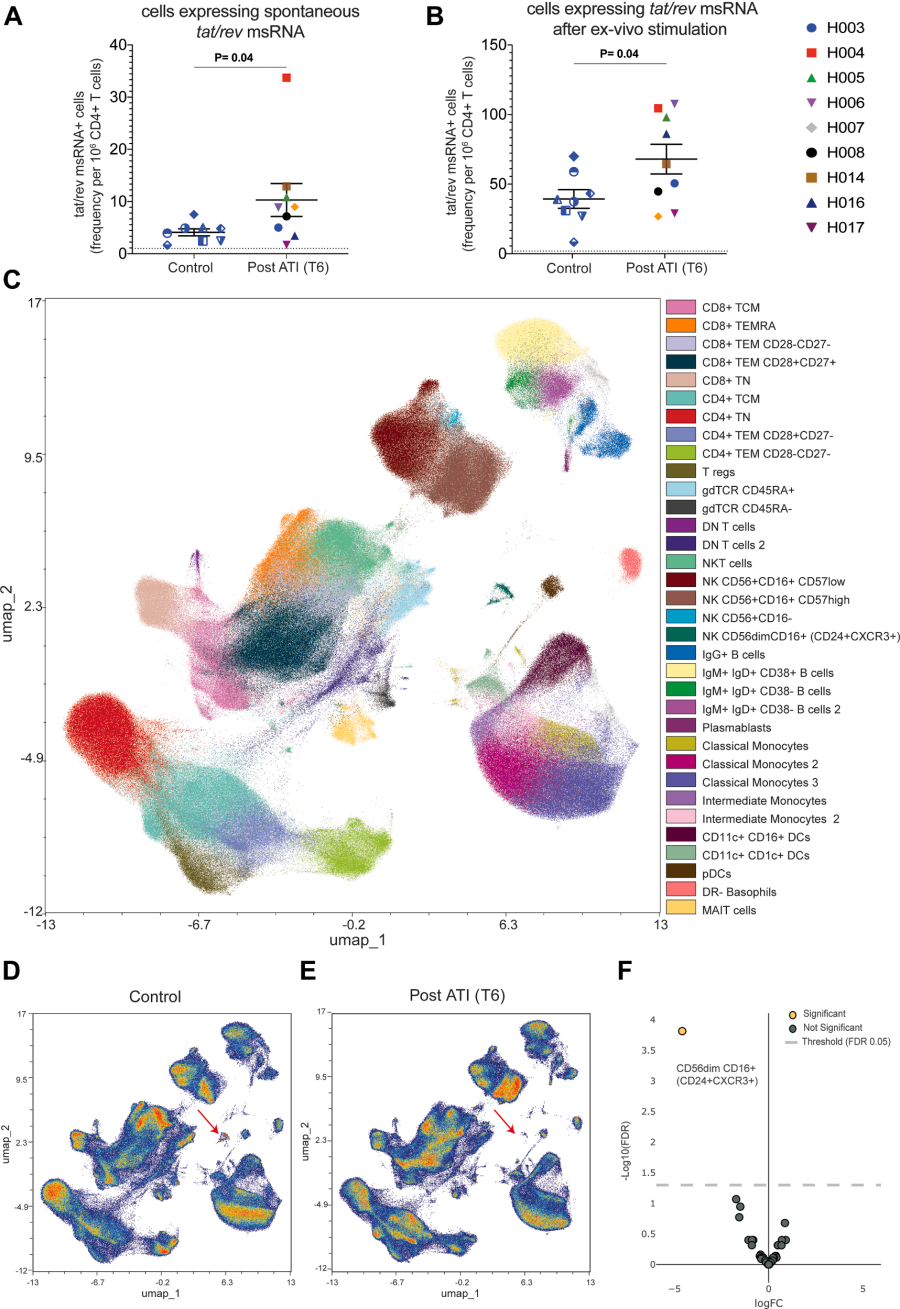
**Inducible HIV-1reservoir and high dimensional flow cytometry analysis of PBMCs from historical controls and post-ATI participants. A**) Comparison of inducible HIV reservoir using samples obtained from historical HIV+ controls (n = 8) and post-ATI participants (n = 9) at timepoint 6. CD4+ T cells spontaneously expressing *tat/rev* msRNA (*in vivo* response) and **B**) inducibly expressing *tat/rev* msRNA after ex vivo treatment with PMA/ionomycin for 12 h, measured using SQuHIVLa. Unpaired t-test was used to compare the means. Two-tailed p-values are shown. **C**) Cell population SPADE clustering of high dimensional 45-colour flow cytometry data visualised with uniform manifold approximation and projection (UMAP) dimensionality reduction. Clusters are annotated based on surface marker expression. The plot shows concatenated files from all samples analysed (n = 17). **D–E**) UMAP plots of data analysed from historical controls (D) and post-ATI participants (E). Red arrow points to a distinct NK cluster CD56dim CD16+ (CD24+CXCR3+) as shown in C. **F**) Volcano plot showing comparison of cluster abundance in historical controls compared to Post-ATI participants as analysed by edgeR. Negative fold change shows decrease in cluster abundance in Post-ATI participants compared to historical controls. Analysis was conducted with an FDR of 0.05.

Since the DC-TRN trial lacked a placebo control, we compared the participants, after more than a decade post-ATI (T6), to a historical cohort of people (n = 8) with HIV-1 subtype B matched for key clinical parameters, including sex, age, CD4+ T cell nadir, CD4+ T cell counts at sampling, and years on ART (Supplementary Table S1). We leveraged PBMC samples from the historical control participants as a comparator group to assess *tat/rev* msRNA expression. Using SQuHIVLa, we observed both a significantly higher frequency of CD4+ T cells spontaneously expressing *tat/rev* msRNA (P = 0.04) as well as significantly higher frequencies of *tat/rev* msRNA expressing cells after ex vivo PMA-ionomycin stimulation for the post ATI participants at T6 compared to the historical control cohort, (P = 0.04) (Fig. 3A and B). These findings support the notion that ATI may leave a durable imprint on the inducible HIV-1 reservoir, even in individuals who re-suppress viraemia and maintain long-term ART. The apparent increase in the inducible HIV-1 reservoir more than a decade post ATI raises important questions about clinical significance and potential role in future viral rebound events.

### In-depth profiling of the immune compartment a decade following ATI reveals discrete signatures in innate and adaptive subsets

While we observed stable viral re-suppression and immune reconstitution (CD4/CD8 ratios) more than a decade after resuming ART (Fig. 1B–D), the long-term impact of vaccination and subsequent extended viral rebound on immune architecture and inflammation remains incompletely understood, especially in context of the observed increase in the inducibility of *tat/rev* msRNA expressing cells. To define whether prolonged ATI leaves an immunological imprint, we performed high-dimensional 45-colour spectral flow cytometry on peripheral blood mononuclear cells (PBMCs) from post-ATI participants at T6 and matched historical HIV+ controls (Supplementary Fig. S2). Initially, manual gating was employed to assess possible changes in key immune populations (Supplementary Fig. S2A). Differential abundance analysis using edgeR showed no statistically significant differences between groups (Supplementary Fig. S2B). To enable a more comprehensive, high-resolution analysis, we used SPADE clustering algorithm and UMAP-based dimensionality reduction to identified discrete immune cell clusters (Supplementary Table S4, Supplementary Fig. S3A) spanning major lymphoid and myeloid compartments (Fig. 3C). UMAP projections revealed no major phenotypic separation between the two groups, except within natural killer (NK) cell subsets (Fig. 3C–E). Volcano plot analysis of cluster abundances using edgeR (FDR <0.05) revealed a marked reduction in the frequency of a distinct population of NK cells in the post-ATI group (Fig. 3F, Supplementary Fig. S3A–E). This population corresponds to a cluster enriched for CD56dim CD16+ NK cells, characterised by differential expression of CD24+ and CXCR3+ relative to other NK cell clusters (Supplementary Fig. S3A–E, Supplementary Table S2).

**Fig. 4:**
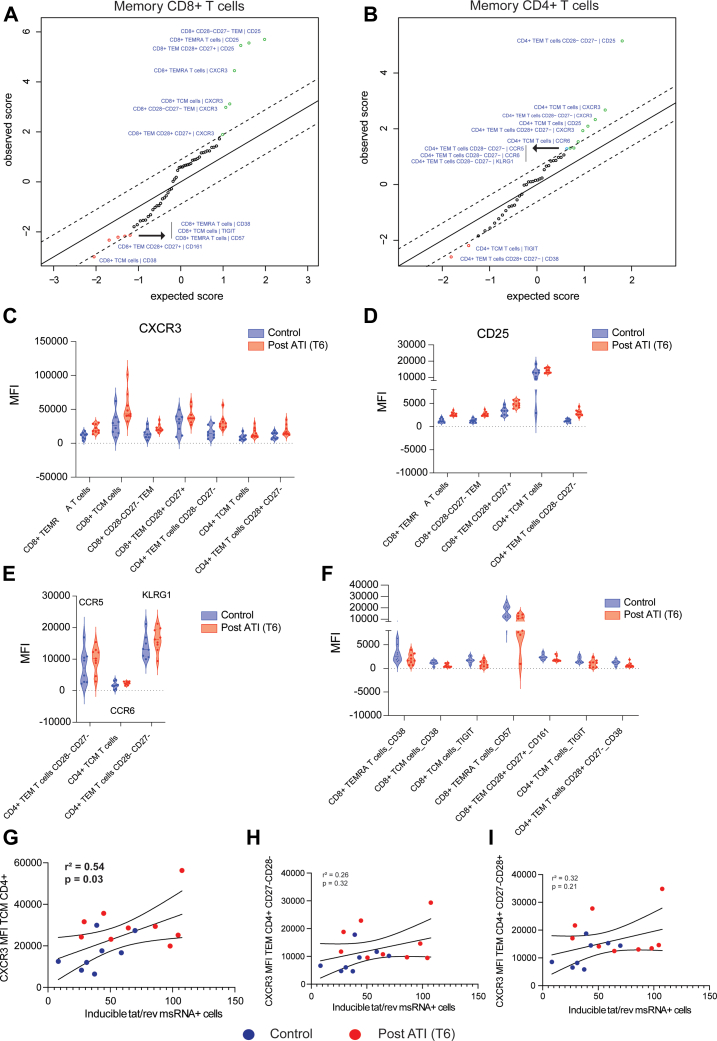
**Multivariate differential analysis of T cell exhaustion and activation marker expression in memory T cell clusters. A–B**) SAM plots depicting differences in median fluorescence intensity of exhaustion and activation markers (CD25, CD38, CD39, HLA-DR, TIGIT, TIM-3, KLRG1, CCR5, CCR6, PD-1, CD57, CXCR3, CXCR5, CD161) in memory CD8+ (A) and CD4+ (B) T cell clusters. The diagonal line represents alignment of observed and expected scores. Coloured points outside of the diagonal line represent a significant increase (green) or decrease (red) in median fluorescence intensity of exhaustion and activation markers of Post-ATI participants at T6 (n = 9) compared to historical HIV+ controls (n = 8). Analysis was conducted with 500 permutations and a FDR of 0.01 to ensure robustness. **C–F**). Violin plots showing mean fluorescence intensity (MFI) values of up- or downregulated activation and exhaustion markers across memory T cell populations identified in the SAM analysis. Plots are grouped based on significant upregulated markers, including CXCR3 (C), CD25 (D), CCR5, CCR6 and KLRG1 (E), as well as downregulated markers (F). **G–I**) Linear regression model for CXCR3 mean fluorescent intensity in TCM CD4+ (G), TEM CD4+ CD27-CD28− (H) and TEM CD4+ CD27-CD28+ (I) clusters and inducible *tat/rev* msRNA+ cells measured by SQuHIVLa. Pearson correlation coefficient and statistical significance was calculated for G (data normally distributed) and Spearman correlation for H and I (data not normally distributed).

We next investigated whether functional phenotypes of memory T cells and NK after ATI were different from historical controls by profiling expression of key exhaustion and activation markers across memory T cell and NK populations in post-ATI (T6) participants and historical HIV+ controls. Supervised multivariate analysis using Significance Analysis of Microarrays (SAM; FDR <0.01) revealed significant differences in the expression pattern of several markers across memory CD8+ and CD4+ T cell subsets (Fig. 4A and B). We observed significant upregulation of marker CXCR3 in memory T cell clusters (Fig. 4) alongside an expansion of CXCR3+ populations (Supplementary Fig. S4). In post-ATI memory CD8+ T cell subsets we also observed an upregulation of T cell activation marker CD25 (Fig. 4D), while expression of C-type lectin receptor CD161 and differentiation marker CD57 was reduced (Fig. 4F), a phenotype previously associated with untreated HIV infection.^47^^,^^48^ In addition, CD4+ memory T cells of post-ATI participants showed similar upregulation of homing and activation markers CXCR3, CCR5, CCR6, KLRG1, and CD25 in effector memory subsets (Fig. 4B, E and F, Supplementary Fig. S4). Interestingly, memory CD8+ and CD4+ T cell subsets in post-ATI participants had a modest but significantly (q-value <0.05) lower expression of exhaustion/chronic activation-associated markers CD38 and TIGIT (Fig. 4F). When analysing NK cell clusters, SAM plots showed no major shift in activation or exhaustion profiles of these subsets, except a moderate downregulation (q-value = 0.22) of markers CD337, CD161, TIGIT, and TIM-3, mostly on CD24+CXCR3+ NK subset in the post-ATI group compared to controls (Supplementary Fig. S5).

Lastly, we set out to investigate whether the phenotypic differences observed in memory CD4+ and CD8+ T cell subsets correlate with HIV-1 reservoir dynamics, prompted by a previous study pointing towards CXCR3+ memory CD4+ T cell subsets as a unique cellular reservoir enriched for inducible replication-competent virus.^49^ Consistent with this data, we observed a significant correlation between CXCR3+ expression on the surface of central memory CD4+ T cells and inducible *tat/rev* msRNA+ cells (Fig. 4G, Supplementary Fig. S6). This data suggests that CXCR3+ expression may serve as a phenotypic biomarker of the inducible HIV-1 reservoir with potential implications for ATI and viral rebound monitoring.

## Discussion

In this study, we leveraged a rare historical human trial cohort to examine whether profound immune perturbation and viral rebound are associated with persistent changes in HIV-1 reservoir characteristics after long-term ART re-suppression. We identify three key observations: (i) higher inducible HIV-1 reservoir activity measured by *tat/rev* msRNA expression despite durable clinical and virologic suppression, (ii) a dissociation between inducible HIV-1 reservoir activity and bulk proviral DNA measures, including total and intact proviral burden, and (iii) differences in memory CD4+ T cell phenotypes, including enrichment of CXCR3-expressing central memory T cell subsets that correlate with inducible HIV-1 *tat/rev* msRNA. These findings extend the prevailing focus on short-term rebound kinetics during treatment interruption by suggesting that rebound-associated perturbation may be linked to longer-term differences in reservoir quality and immune architecture long after ART-mediated suppression is re-established. This interpretation is also consistent with the delayed normalisation of the CD4+/CD8+ T cell ratio after ART resumption observed in our cohort. Similar findings have been reported after treatment interruption in children, where persistent elevation of CD8+ T cells and slower recovery of the CD4/CD8 ratio were observed after ART re-initiation relative to continuous therapy controls.^50^ In adults, shorter-term data after repeated ATI also suggest that CD8+ T-cell counts may remain above baseline despite ART resumption.^51^ Together, these findings suggest that viral re-suppression and recovery of CD4+ T cell counts may coexist with incomplete normalisation of the CD8+ T-cell compartment after ATI.

A key implication of our work is that functional HIV-1 reservoir properties may shift without proportionate changes in the size of the proviral reservoir as captured by commonly used DNA-based endpoints. However, this interpretation requires caution because paired measurements of proviral DNA and *gag/pol* vRNA at baseline and T1/T2 were not feasible in this study. Particularly, the FISH-Flow assay requires a substantial input of isolated CD4+ T cells, approximately 10–20 million cells per condition, which was not available for these samples. In addition, even under stimulated conditions, *gag/pol* vRNA+ cells were detected at low frequencies, with a median of 18 cells per million CD4+ T cells (IQR: 13–40), suggesting that unstimulated *gag/pol* vRNA+ events would likely fall close to or below the assay's limit of detection (∼6 *gag/pol* vRNA+ cells per million CD4+ T cells).^40^ Therefore, we cannot determine whether increased inducible *tat/rev* msRNA was accompanied by parallel changes in later viral transcriptional products or by changes in the overall frequency of HIV DNA-positive cells under the same stimulation conditions. This limitation is important because total HIV DNA *gag/pol* vRNA, and inducible *tat/rev* msRNA capture related but distinct dimensions of the reservoir: total HIV DNA reflects overall proviral burden,^52^ *gag/pol* vRNA reflects later or more extensive viral transcription, whereas *tat/rev* msRNA may reflect early/*rev*-dependent transcriptional activity and inducibility (reviewed in 53, 54, 55). Within this context, our observed increase in inducible *tat/rev* msRNA despite minimal change in total or intact proviral DNA may indicate altered inducibility of existing reservoir and not expansion of the total proviral reservoir. Moreover, HIV-1 persistence is sustained by a heterogeneous reservoir in which defective proviruses are abundant, limiting the interpretability of bulk proviral quantification alone. Increased inducible *tat/rev* msRNA may thus reflect altered transcriptional competence, chromatin context, integration site distribution, or clonal expansion of infected cells –rather than simply expanding overall proviral frequency.^41^, ^42^, ^43^^,^^53^^,^^54^ These findings reinforce the broader concept that reservoir activity is governed not only by quantity but also by qualitative features that determine the propensity to reactivate under immune and inflammatory signals. Indeed, earlier studies by Fischer et al. demonstrated *tat/rev* msRNA expression concomitantly occurring with the re-emergence of PBMC-associated HIV-1 particles (i.e., cellular viral rebound) after two weeks of structured ART interruption, which was predicted by during therapy levels of *nef* msRNA transcripts in PBMCs.^56^ More recently, De Scheerder et al., showed an increase in *tat/rev* RNA transcripts, amongst elevated expression of restriction factors (SLFN11 and APOBEC3G), after ATI prior to viral rebound, indicating the use of these restriction factors and *tat/rev* RNA as potential biomarkers for imminent viral rebound.^14^ However, our exploratory analyses suggest that different reservoir metrics may capture distinct aspects of rebound risk, with baseline total HIV DNA and post-vaccination intact HIV DNA frequency associating with faster rebound, whereas *tat/rev* msRNA-based measures showed less straight forward relationships (Supplementary Fig. S7). Overall, these findings should be interpreted cautiously given the small sample size, the absence of paired pre- and post-stimulation *gag/pol* vRNA and total HIV DNA measurements, and the fact that some measures were obtained after vaccination rather than at baseline. While *tat/rev* msRNA-based measures may capture reservoir state and inducibility, their predictive value for imminent viral rebound remains to be established in larger longitudinal ATI studies incorporating paired measurements of proviral DNA, *gag/pol* vRNA, inducible *tat/rev* ms RNA and viral rebound kinetics.

High dimensional spectral flow cytometry and unsupervised clustering revealed broadly similar immune-subset distributions between post-ATI participants and controls consistent with overall immune reconstitution. However, we did observe a significantly lower frequency of a CD56dim CD16+ NK cluster in samples from trial participants compared to control samples. This cluster was enriched for CD56dim CD16+ NK cells that differentially express high levels of markers CD24+ and CXCR3+ compared to other NK cell clusters. The elevated expression of CXCR3+ suggests a migratory phenotype within this population.^57^ Interestingly, CD24 is a characteristic marker of B cells and is not typically expressed on NK cells, making this cluster phenotypically distinct. As the functional characteristics and biological relevance of this population are unclear, further studies are warranted to clarify its role in the post-viral rebound immune landscape. In a previous study investigating the impact of DC vaccination on NK cells in the DCTRN trial participants,^58^ Laeremans et al., did not observe significant changes in the frequencies of total NK cells across vaccination and ATI timepoints (PreVac: prior to vaccination (baseline); Vac2 + w1: 1 week after the second vaccination; ATI + 4w and ATI + 16w: 4 or 16 weeks after ATI). However, a significant decrease in the CD56dimCD16^−^ subset together with an increase in the CD56dimCD16+ subset was observed at Vac2 + 1w compared to PreVac. The frequencies of the CD56dimCD16− and CD56dimCD16+ subsets did not return to PreVac levels after ATI, suggesting a sustained impact of vaccination and viral rebound on the distribution of these NK cell subsets. Due to limited quantities or unavailable baseline PBMCs in the present long-term analysis, we could not determine whether early vaccine or viral rebound-associated shifts in CD56dimCD16− and CD56dimCD16+ NK cells persisted through ART resumption and after long-term viral re-suppression. In addition, direct comparison across studies is constrained by non-overlapping time points, marker panels, and differences in comparator groups (HIV-1 negative controls in Laereman's study versus chronic HIV-1 on long term, uninterrupted ART, here).

Within CD4+ and CD8+ memory T cells, we observed a complex phenotypic profile: increased surface expression of homing and activation markers (CCR5, CCR6, CXCR3, CD25, KLRG1), decreased expression of mucosal marker CD161 and differentiation marker CD57, and a partial reduction in classical exhaustion and chronic activation markers (TIGIT, CD38) at T6. Similar shifts in CXCR3, CD161 and CD57 expression have been reported in progressive HIV infection and in cancer, where they are associated with altered effector function and immune dysregulation.^47^^,^^59^^,^^60^ A decreased expression of TIGIT and CD38 at T6 indicates that the pattern we observe is not consistent with a classical profile of chronic activation or inflammation but rather suggests a state characterised by enhanced trafficking (CXCR3) and pro-inflammatory marker upregulation (CD25, CCR6, CCR5). However, further assays are needed to determine if these phenotypic changes present in the post-ATI participants associate with a functional phenotype, and whether they are reproducible across other post-ATI cohorts.

Notably, among the differentially expressed markers, the activation and tissue homing receptor CXCR3 was highly upregulated in both memory CD4+ and CD8+ T cell subsets of post-ATI participants, as reflected by elevated CXCR3 expression and higher frequency of CXCR3+ cells. CXCR3 is a chemokine receptor expressed on T cells upon activation and plays a crucial role in trafficking and effector responses of memory T cells.^61^ In people with HIV-1, CXCR3 expression is increased in circulating memory CD4+ and CD8+ T cells and gut mucosal CD4+ T cells compared to healthy individuals.^62^^,^^63^ In our cohort, CXCR3 upregulation may indicate increased tissue homing driven by persistent inflammatory cues, even in the presence of long-term viral suppression. CXCR3 expression on central memory CD4+ T cells positively correlates with inducible *tat/rev* msRNA expression, whereas this correlation was not observed in effector memory subsets or in CD8+ T cells. Given that central memory CD4+ T cells represent a reservoir niche,^64^ this selective association suggests that CXCR3 may mark a reservoir-relevant subset enriched for inducible provirus, consistent with recent evidence that circulating CXCR3+ memory CD4+ T cells are enriched for inducible replication-competent provirus.^49^ Together with prior observations in this study population that CXCR3 ligands increase during HIV-1 reactivation during ATI,^65^ our data support the further evaluation of the CXCR3 axis as a candidate link between tissue trafficking programs to reservoir inducibility and may serve as a practical biomarker target for monitoring reservoir dynamics during HIV-1 cure interventions.

From a design and translational standpoint, and aligned with the rationale for deeper monitoring, our study supports integrating inducible HIV reservoir assays, such as SQuHIVLa, with high-dimensional multiparameter immunophenotyping to capture impact of ATI beyond plasma HIV-1 RNA levels and CD4+ T cell counts. However, several limitations merit discussion. Firstly, the primary clinical protocol required participants to restart ART when a participant's CD4+ T cell count decreased below 50% of the baseline value at study entry or when re-treatment was considered necessary according to clinical guidelines at the time. The trial monitored participants closely up to week 96 after ATI to determine how long they could safely remain off ART.^29^ The prolonged ATI protocol in the DC-TRN trial thus reflects an earlier era of therapeutic vaccination studies and does not align with current, shorter ATI paradigms and contemporary safeguards^23^^,^^25^, ^26^, ^27^; recent consensus recommendations restrict prolonged ATI to carefully justified settings with intensive monitoring and predefined ART resumption criteria.^66^ Secondly, the sample size is small, owing to the rarity of long-term ART-treated ATI participants with suitable archived longitudinal samples, and the analyses may be underpowered to detect smaller between-group or subgroup differences. Furthermore, immunophenotypic comparison between T6 and T0 was not feasible, as the T0 samples consisted of aliquots of the non-adherent cell fraction retained during DCTRN autologous vaccine preparation, rather than unprocessed whole PBMCs. These samples were therefore not appropriate for whole-PBMC phenotypic or inducible viral reservoir analyses. The use of historical comparators, although carefully matched, cannot fully account for unmeasured confounding and does not provide concomitant reservoir measurements for all endpoints. Because the parent DC-TRN trial included only male participants, the present findings may not be generalisable to female participants. Future ATI studies should include more diverse cohorts to assess whether sex-related immunological differences influence reservoir inducibility, immune remodelling, and rebound kinetics. In addition, the study size was determined by the availability of suitable archived samples and feasibility of additional leukapheresis sampling rather than by a formal power calculation; therefore, these secondary analyses should be interpreted as exploratory and hypothesis-generating. Given that the intervention occurred many years earlier, dense longitudinal sampling across ATI and the immediate post-ART resumption period was not available, limiting our ability to resolve the temporal sequence of immune remodelling versus HIV-1 reservoir inducibility changes. Notably, prior transcriptome analyses in this study population showed that vaccination and viral rebound during ATI were associated with substantial systemic transcriptional changes,^30^ which is consistent with the long-term phenotypic differences observed here. Lastly, although our analyses focused on accessible blood compartments, tissue-based reservoirs remain central to HIV-1 persistence, and we cannot determine whether the observed CXCR3 upregulation reflects distribution among tissues, altered immune surveillance, or changes intrinsic to infected clones. Future prospective studies incorporating dense sampling across ATI phases, tissue compartments, and functional readouts will be needed to test these mechanistic hypotheses.

In conclusion, our study suggests that profound immune perturbation and viral rebound may be associated with persistent changes in HIV-1 reservoir inducibility and immune architecture even after long-term ART re-suppression. The observed increase in inducible HIV-1 reservoir activity (marked by *tat/rev* msRNA), in the absence of parallel increases in total or intact proviral DNA, together with associations between CXCR3+ central memory CD4+ T cell phenotypes and HIV-1 inducibility, highlights functional reservoir metrics and immune trafficking programmes as candidate biomarkers and mechanistic tools for HIV-1 cure research. These findings support further evaluation of immune and reservoir monitoring beyond short-term viral rebound windows, particularly in studies involving repeated or prolonged analytical treatment interruptions. They also underscore the importance of clearly communicating both short-term and potential longer-term risks during informed consent processes. In this context, ART-resumption criteria should be viewed not merely as operational trial rules, but as core ethical safeguards that shape cumulative exposure to viraemia and its potential consequences including immune perturbation, reservoir remodelling, clinical symptoms, transmission risk, and psychosocial burden. Overall, our findings support a framework in which ATI remains an important research tool, but one that should be paired with careful immunovirological surveillance and further evaluation of candidate biomarkers that may inform risk mitigation in future studies.

## Contributors

Conceptualisation: C.L, and T.Ma; Methodology: C.L., T.H., RC., Y.M.M, and T.Ma; Investigation: C.L., T.H., R.C., H.A.B.P., K.S.H.; Data Curation and Formal Analysis: C.L., T.H., R.C., and T.Ma; Validation, C.L., T.H., R.C., and T.Ma; Data Visualisation: C.L., T.H., R.C., and T.Ma.; Project Administration: C.L., R.C., H.A.B.P., K.S.H., and T.Ma; Supervision: R.A.G., C.R., Y.M.M., and T.Ma; Funding Acquisition, C.L., S.R., C.R., R.A.G., Y.M.M., and T.Ma.; Resources: C.L., S.R., C.R., R.A.G., Y.M.M., and T.Ma; Writing—Original draft, C.L. T.H., and R.C.; Writing—Review and Editing, C.L., T.H., R.C., H.A.B.P., K.S.H., C.R., S.R., J.J.A.vK., D.A.M.C.V., T.M., P.D.K., Y.M.M., R.A.G., and T.Ma. C.L. RC, and T.Ma directly accessed and verified the underlying data reported in the manuscript. All authors read and approved the final version of the manuscript.

## Data sharing statement

The authors can share the presented and additional data from this study upon request. Due to participant privacy concerns and consent limitations, individual-level demographic and clinical data cannot be publicly shared. For further information, please contact: r.gruters@erasmusmc.nl; t.mahmoudi@erasmusmc.nl.

## Declaration of interests

C.L is currently employed by Aidsfonds since April 2026. K.S.H received support from Gilead for attending the *HIV Glasgow* meeting and support from ViiV Healthcare for attending an Expert–Expert meeting, paid to institution. S.R. is currently employed by ViiV Healthcare since June 2025. D.A.M.C.V. received grants from ViiV Healthcare, Gilead Sciences, NIH and NWO, paid to institution; Consulting fees from ViiV healthcare, paid to institution, and support from Gilead Sciences for attending meetings. C.R. received Research grants IIS from ViiV Healthcare and Gilead, paid to institution. C.R received support from Gilead and ViiV Healthcare for attending meetings, paid to institution. C.R. has received support from Gilead, ViiV and MSD for serving on Data Safety Monitoring and Advisory Boards and holds stocks in Immunocore and Eli Lilly. All other authors declare no competing interests.

## References

[1] Chun T.W., Carruth L., Finzi D. (1997). Quantification of latent tissue reservoirs and total body viral load in HIV-1 infection. Nature.

[2] Chun T.W., Stuyver L., Mizell S.B. (1997). Presence of an inducible HIV-1 latent reservoir during highly active antiretroviral therapy. Proc Natl Acad Sci U S A.

[3] Siliciano J.D., Siliciano R.F. (2006). The latent reservoir for HIV-1 in resting CD4+ T cells: a barrier to cure. Curr Opin HIV AIDS.

[4] Finzi D., Blankson J., Siliciano J.D. (1999). Latent infection of CD4+ T cells provides a mechanism for lifelong persistence of HIV-1, even in patients on effective combination therapy. Nat Med.

[5] Joos B., Fischer M., Kuster H. (2008). HIV rebounds from latently infected cells, rather than from continuing low-level replication. Proc Natl Acad Sci U S A.

[6] Hodel F., Patxot M., Snäkä T., Ciuffi A. (2016). HIV-1 latent reservoir: size matters. Future Virol.

[7] Bruner K.M., Wang Z., Simonetti F.R. (2019). A quantitative approach for measuring the reservoir of latent HIV-1 proviruses. Nature.

[8] Ho Y.C., Shan L., Hosmane N.N. (2013). Replication-competent noninduced proviruses in the latent reservoir increase barrier to HIV-1 cure. Cell.

[9] Estes J.D., Kityo C., Ssali F. (2017). Defining total-body AIDS-virus burden with implications for curative strategies. Nat Med.

[10] Cohn L.B., Chomont N., Deeks S.G. (2020). The biology of the HIV-1 latent reservoir and implications for cure strategies. Cell Host Microbe.

[11] Deeks S.G., Lewin S.R., Ross A.L. (2016). International AIDS Society global scientific strategy: towards an HIV cure 2016. Nat Med.

[12] Deeks S.G., Archin N., Cannon P. (2021). Research priorities for an HIV cure: international AIDS society global scientific strategy 2021. Nat Med.

[13] Gunst J.D., Gohil J., Li J.Z. (2025). Time to HIV viral rebound and frequency of post-treatment control after analytical interruption of antiretroviral therapy: an individual data-based meta-analysis of 24 prospective studies. Nat Commun.

[14] De Scheerder M.A., Van Hecke C., Zetterberg H. (2020). Evaluating predictive markers for viral rebound and safety assessment in blood and lumbar fluid during HIV-1 treatment interruption. J Antimicrob Chemother.

[15] Papasavvas E., Lada S.M., Joseph J. (2018). Analytical antiretroviral therapy interruption does not irreversibly change preinterruption levels of cellular HIV. AIDS.

[16] Scutari R., Costabile V., Galli L. (2021). Impact of analytical treatment interruption on burden and diversification of HIV peripheral reservoir: a pilot study. Viruses.

[17] Salantes D.B., Zheng Y., Mampe F. (2018). HIV-1 latent reservoir size and diversity are stable following brief treatment interruption. J Clin Invest.

[18] Strongin Z., Sharaf R., VanBelzen D.J. (2018). Effect of short-term antiretroviral therapy interruption on levels of integrated HIV DNA. J Virol.

[19] Clarridge K.E., Blazkova J., Einkauf K. (2018). Effect of analytical treatment interruption and reinitiation of antiretroviral therapy on HIV reservoirs and immunologic parameters in infected individuals. PLoS Pathog.

[20] Gantner P., Buranapraditkun S., Pagliuzza A. (2023). HIV rapidly targets a diverse pool of CD4(+) T cells to establish productive and latent infections. Immunity.

[21] Chun T.W., Engel D., Berrey M.M., Shea T., Corey L., Fauci A.S. (1998). Early establishment of a pool of latently infected, resting CD4(+) T cells during primary HIV-1 infection. Proc Natl Acad Sci U S A.

[22] Colby D.J., Trautmann L., Pinyakorn S. (2018). Rapid HIV RNA rebound after antiretroviral treatment interruption in persons durably suppressed in Fiebig I acute HIV infection. Nat Med.

[23] Lau J.S.Y., Smith M.Z., Lewin S.R., McMahon J.H. (2019). Clinical trials of antiretroviral treatment interruption in HIV-infected individuals. Aids.

[24] Strategies for Management of Antiretroviral Therapy Study G., El-Sadr W.M., Lundgren J. (2006). CD4+ count-guided interruption of antiretroviral treatment. N Engl J Med.

[25] Wen Y., Bar K.J., Li J.Z. (2018). Lessons learned from HIV antiretroviral treatment interruption trials. Curr Opin HIV AIDS.

[26] Zheng L., Tierney C., Bosch R.J. (2021). Analytical treatment interruption in HIV trials: statistical and study design considerations. Curr HIV/AIDS Rep.

[27] Alexandre M., Prague M., Lhomme E. (2024). Definition of virological endpoints improving the design of HIV cure strategies using analytical antiretroviral treatment interruption. Clin Infect Dis.

[28] Montserrat M., Plana M., Guardo A.C. (2017). Impact of long-term antiretroviral therapy interruption and resumption on viral reservoir in HIV-1 infected patients. AIDS.

[29] Allard S.D., De Keersmaecker B., de Goede A.L. (2012). A phase I/IIa immunotherapy trial of HIV-1-infected patients with Tat, Rev and Nef expressing dendritic cells followed by treatment interruption. Clin Immunol.

[30] de Goede A.L., Andeweg A.C., van den Ham H.J. (2015). DC immunotherapy in HIV-1 infection induces a major blood transcriptome shift. Vaccine.

[31] van Snippenberg W., Gleerup D., Rutsaert S., Vandekerckhove L., De Spiegelaere W., Trypsteen W. (2022). Triplex digital PCR assays for the quantification of intact proviral HIV-1 DNA. Methods.

[32] Sarabia I., Huang S.H., Ward A.R., Jones R.B., Bosque A. (2021). The intact non-inducible latent HIV-1 reservoir is established in an in vitro primary T(CM) cell model of latency. J Virol.

[33] Baxter A.E., Niessl J., Fromentin R. (2017). Multiparametric characterization of rare HIV-infected cells using an RNA-flow FISH technique. Nat Protoc.

[34] Rao S., Lungu C., Crespo R. (2021). Selective cell death in HIV-1-infected cells by DDX3 inhibitors leads to depletion of the inducible reservoir. Nat Commun.

[35] Hossain T., Lungu C., de Schrijver S. (2024). Specific quantification of inducible HIV-1 reservoir by RT-LAMP. Commun Med.

[36] Lungu C., Procopio F.A. (2022). TILDA: Tat/Rev induced limiting dilution assay. Methods Mol Biol.

[37] Park L.M., Lannigan J., Low Q., Jaimes M.C., Bonilla D.L. (2024). OMIP-109: 45-color full spectrum flow cytometry panel for deep immunophenotyping of the major lineages present in human peripheral blood mononuclear cells with emphasis on the T cell memory compartment. Cytometry A.

[38] Qiu P., Simonds E.F., Bendall S.C. (2011). Extracting a cellular hierarchy from high-dimensional cytometry data with SPADE. Nat Biotechnol.

[39] Healy J., McInnes L. (2024). Uniform manifold approximation and projection. Nat Rev Methods Primers.

[40] Sannier G., Dube M., Dufour C. (2021). Combined single-cell transcriptional, translational, and genomic profiling reveals HIV-1 reservoir diversity. Cell Rep.

[41] Einkauf K.B., Lee G.Q., Gao C. (2019). Intact HIV-1 proviruses accumulate at distinct chromosomal positions during prolonged antiretroviral therapy. J Clin Invest.

[42] Einkauf K.B., Osborn M.R., Gao C. (2022). Parallel analysis of transcription, integration, and sequence of single HIV-1 proviruses. Cell.

[43] Siliciano J.D., Siliciano R.F. (2021). Low inducibility of latent human immunodeficiency virus type 1 proviruses as a major barrier to cure. J Infect Dis.

[44] Grau-Expósito J., Luque-Ballesteros L., Navarro J. (2019). Latency reversal agents affect differently the latent reservoir present in distinct CD4+ T subpopulations. PLoS Pathog.

[45] Debrabander Q., Hensley K.S., Psomas C.K. (2023). The efficacy and tolerability of latency-reversing agents in reactivating the HIV-1 reservoir in clinical studies: a systematic review. J Virus Erad.

[46] Martin H.A., Kadiyala G.N., Telwatte S. (2022). New assay reveals vast excess of defective over intact HIV-1 transcripts in antiretroviral therapy-suppressed individuals. J Virol.

[47] Lee S.A., Sinclair E., Hatano H. (2014). Impact of HIV on CD8+ T cell CD57 expression is distinct from that of CMV and aging. PLoS One.

[48] Gaardbo J.C., Hartling H.J., Thorsteinsson K., Ullum H., Nielsen S.D. (2013). CD3+CD8+CD161high Tc17 cells are depleted in HIV-infection. AIDS.

[49] Banga R., Procopio F.A., Ruggiero A. (2018). Blood CXCR3(+) CD4 T cells are enriched in inducible replication competent HIV in aviremic antiretroviral therapy-treated individuals. Front Immunol.

[50] Freguja R., Bamford A., Zanchetta M. (2021). Long-term clinical, virological and immunological outcomes following planned treatment interruption in HIV-infected children. HIV Med.

[51] Jain A., Canepa G.E., Liou M.L. (2024). Multiple treatment interruptions and protecting HIV-specific CD4 T cells enable durable CD8 T cell response and viral control. Front Med (Lausanne).

[52] Avettand-Fenoel V., Hocqueloux L., Ghosn J. (2016). Total HIV-1 DNA, a marker of viral reservoir dynamics with clinical implications. Clin Microbiol Rev.

[53] Fombellida-Lopez C., Berkhout B., Darcis G., Pasternak A.O. (2024). Persistent HIV-1 transcription during ART: time to reassess its significance?. Curr Opin HIV AIDS.

[54] Pasternak A.O., Berkhout B. (2023). HIV persistence: silence or resistance?. Curr Opin Virol.

[55] Murzin A.I., Elfimov K.A., Gashnikova N.M. (2024). The proviral reservoirs of Human Immunodeficiency Virus (HIV) infection. Pathogens.

[56] Fischer M., Joos B., Hirschel B. (2004). Cellular viral rebound after cessation of potent antiretroviral therapy predicted by levels of multiply spliced HIV-1 RNA encoding nef. J Infect Dis.

[57] Ran G.H., Lin Y.Q., Tian L. (2022). Natural killer cell homing and trafficking in tissues and tumors: from biology to application. Signal Transduct Target Ther.

[58] Laeremans T., den Roover S., Lungu C. (2023). Autologous dendritic cell vaccination against HIV-1 induces changes in natural killer cell phenotype and functionality. NPJ Vaccines.

[59] Brainard D.M., Tager A.M., Misdraji J. (2007). Decreased CXCR3+ CD8 T cells in advanced human immunodeficiency virus infection suggest that a homing defect contributes to cytotoxic T-lymphocyte dysfunction. J Virol.

[60] Perdomo-Celis F., Taborda N.A., Rugeles M.T. (2019). CD8(+) T-Cell response to HIV infection in the era of antiretroviral therapy. Front Immunol.

[61] Groom J.R., Luster A.D. (2011). CXCR3 in T cell function. Exp Cell Res.

[62] Olivo A., Lécuroux C., Bitu M. (2021). CXCR3 and CXCR5 are highly expressed in HIV-1-specific CD8 central memory T cells from infected patients. Eur J Immunol.

[63] Loiseau C., Requena M., Nayrac M. (2019). Increased CXCR3+ T cells impairs recruitment of T-Helper type 17 cells via interferon γ and interleukin 18 in the small intestine mucosa during treated HIV-1 infection. J Infect Dis.

[64] Lee G.Q., Lichterfeld M. (2016). Diversity of HIV-1 reservoirs in CD4+ T-cell subpopulations. Curr Opin HIV AIDS.

[65] van den Ham H.J., Cooper J.D., Tomasik J. (2018). Dendritic cell immunotherapy followed by cART interruption during HIV-1 infection induces plasma protein markers of cellular immunity and neutrophil recruitment. PLoS One.

[66] Ndung'u T., Dong K.L., Colby D.J. (2026). Recommendations from the 2nd consensus workshop on analytical treatment interruption in HIV research trials. Lancet HIV.

