# Supplementary material for: HIV-1 reservoir inducibility and immune phenotypes after prolonged analytical treatment interruption: a long-term follow-up study
Source: eBioMedicine. 2026 Aug 1;131:106411. doi: 10.1016/j.ebiom.2026.106411 (PMC13453597; doi:10.1016/j.ebiom.2026.106411)
Supplement: Supplementary file 1 [file mmc1.docx]

**Supplementary data**

**HIV-1 reservoir inducibility and immune phenotypes after prolonged analytical treatment interruption: a long-term follow-up study**

**Supplementary Fig. 1. Longitudinal HIV-1 DNA dynamics.** Pie charts of intact and defective HIV-1 DNA (5’ Deleted [5’Del] and 3’ Deleted/Hypermutated [3’Del/Hyper]) are shown for each participant. The proportions of intact and defective DNA are shown as percentages of total HIV DNA copies/million CD4+ T cells. Intact HIV DNA was distinguished from defective HIV DNA using the intact proviral DNA assay (IPDA). N/A = not available.

**Supplementary Fig. 2.** **OMIP-109** **Gating Strategy.** **A**) Manual gating strategy for flow cytometric analysis of PBMC populations. **B**) Volcano plot showing comparison of population abundance (as gated in A) in historical HIV+ controls compared to post-ATI participants as analysed by edgeR. Analysis was conducted with an FDR of 0.05.

**Supplementary Fig. 3**. **Immune Cell Subset Clustering**. **A**) Clustered heatmap of medians for 43 features (excl. CD45 and Annexin V as cells were subsampled from the live CD45+ population) for all clusters defined by SPADE (Fig. 3). All 17 samples were concatenated and relative median fluorescence expression was used to annotate the different clusters. **B-E)** Histogram overlays of CD16 (B), CD56 (C), CD24 (D) and CXCR3 (E) in distinct NK populations in concatenated samples.

**Supplementary Fig. 4. A**) Histograms of marker expression for all cells (all clusters) and all samples (17 files overlaid) for markers characterized in Figure 4C-F. Positivity threshold is defined based on the distribution of expression using internal negative populations and gates are placed above this baseline population. **B-E**) Percentage of cells in the specified cluster expressing CXCR3 (B), CD25 (C), CCR5, CCR6 or KLRG1 (D), CD38, TIGIT, CD57 or CD161 (E). Positive populations are defined as explained above (A). Thresholds were applied uniformly across all samples and clusters. Statistical significance was assessed using two-way ANOVA for repeated measures (*p<0.05, **p<0.01, ***p<0.001).

**Supplementary Fig. 5**. **Analysis of exhaustion and activation marker expression in NK cells.** **A**) SAM plot shows changes in median fluorescence intensity of surface markers PD-1, TIM-3, KLRG1, TIGIT, CD161 and CD337 in NK cell clusters. The diagonal line represents alignment of observed and expected scores. Red points outside of the line represent a significant decrease (red) in median fluorescence intensity of said markers.

**Supplementary Fig.6.** **Relationship between T cell subsets expressing CXCR3 and inducible *tat/rev* msRNA+ cells**. **A-D**) Linear regression model for CXCR3 mean fluorescence intensity in TCM CD8+ (A), TEM CD8+ CD27-CD28- (B), TEM CD4+ CD27-CD28+ (C), TEMRA CD8+ (D) clusters and inducible *tat/rev* msRNA as measured by SQuHIVLa. Correlation coefficients and statistical significance were calculated using Pearson correlation as the data were normally distributed.

**Supplementary Fig.7. Exploratory associations between reservoir measures and rebound kinetics after ATI**. **A–C**) Correlations between baseline, pre-vaccination (T0) reservoir measures and time to viral rebound, defined as plasma HIV-1 RNA >1000 copies/mL. Shown are total HIV DNA (A), intact HIV DNA expressed as copies per million CD4+ T cells (B), and intact HIV DNA frequency (%) (C). **D–F**) Correlations between post-vaccination (T2) reservoir measures and time to viral rebound >1000 copies/mL, including total HIV DNA (D), intact HIV DNA expressed as copies per million CD4+ T cells (E), and intact HIV DNA frequency (%) (F). **G–I**) Correlations between pre-ATI transcriptional reservoir measures and viral rebound kinetics. Shown are pre-ATI unspliced *gag/pol* vRNA+ cells versus time to viral rebound >1000 copies/mL (G), and spontaneous *tat/rev* msRNA+ cells versus time to first detectable viraemia >50 copies/mL (H) or time to viral rebound >1000 copies/mL (I). Each symbol represents one participant. Spearman correlation coefficients (r) and two-sided P values are shown in the corresponding panels.

**Supplementary Table 1. Post-ATI and historical cohort characteristics**

*DC-TRN trial participants included in this study

|  | **Control cohort** | **Post-ATI cohort*** | **p-value** |
| --- | --- | --- | --- |
| Sex assigned at birth, n | Male, 8 | Male, 9 | - |
| Age at ART initiation, median (IQR) | 40.5 (40.2-49.2) | 38 (32.5-43.0) ^a^ | **0.04** |
| Plasma VL at ART initiation (log10 c/mL) | 4.8 (4.1-5.7) | 5.0 (4.9 -5.0) ^a^ | 0.35 |
| Nadir CD4+ T cell count, median (IQR) | 225 (65-420) | 250 (180-300) | 0.92 |
| Time to VL suppression after ART initiation in months, median (IQR) | 5.0 (5-13.5) | 6.5 (6.0-7.8) ^a^ | 0.43 |
| Years on ART, median (IQR) | 9.9 (7.1-18.3) | 12.4 (11.9-13.5) ^b^ | 0.88 |
| Years on ART VL <50 c/mL, median (IQR) | 7.6 (5.8-8.5) | 10.8 (7.8-12.0) ^b^ | 0.26 |
| Age at sampling, median (IQR) | 57 (52-65) | 60 (57-68) ^c^ | 0.42 |
| Last known CD4+ T cell counts (cells/mm^3^) around time of sampling, median (IQR) | 665 (513-903) | 590 (490-780) ^c^ | 0.38 |

^a^ at first initiation

^b^ after ATI and ART resumption

^c^ after ATI and ART resumption (Time point 6)

ART: Antiretroviral therapy; IQR: Interquartile range; VL: viral load; c/mL: copies/mL; ATI: Analytical treatment interruption

| **Amplicon** | **Primer/probe** | **Sequence (5’-3’)** |
| --- | --- | --- |
| PSI | Forward primer | CAGGACTCGGCTTGCTGAAG |
| PSI | Reverse primer | GCACCCATCTCTCTCCTTCTAGC |
| PSI | Probe | /56-FAM/TTTTGGCGT/ZEN/ACTCACCAGT/3IABkFQ/ |
| *env* | Forward primer | AGTGGTGCAGAGAGAAAAAAGAGC |
| *env* | Reverse primer | GTCTGGCCTGTACCGTCGC |
| *env* | Probe | /5HEX/CCTTGGGTT/ZEN/CTTGGGA/3IABkFQ/ |
| *RPP30* | Forward primer | GCTAACCTTTGATAGGCTCAGT |
| *RPP30* | Reverse primer | CAAATGGTAAGAAGGGCAGAAAC |
| *RPP30* | Probe | /56-FAM/AGCTAAGTG/ZEN/TTGATCCATCTCTTTCTAGCT/AbKFQ |
| *RPP30-2* | Forward primer | AGATTTGGACCTGCGAGCG |
| *RPP30-2* | Reverse primer | GAGCGGCTGTCTCCACAAGT |
| *RPP30-2* | Probe | /HEX/TTCTGACCT/ZEN/GAAGGCTCTGCGCG/AbKFQ |

**Supplementary Table 2.** **Intact Proviral DNA Assay Primers and Probes**

**Supplementary Table 3. Antibody reagents used for high-dimensional flow cytometry**

| **Specificity** | **Fluorochrome** | **Clone** | **Catalog #** | **Vendor** | **RRID** |
| --- | --- | --- | --- | --- | --- |
| CD1c | Alexa Fluor 647 | L161 | 331510 | BioLegend | AB_1186032 |
| CD2 | PerCP/Cy5.5 | TS1/8 | 309225 | BioLegend | AB_2687220 |
| CD3 | BV510 | SK7 | 344827 | BioLegend | AB_2563703 |
| CD4 | PerCP/Fire 806 | SK3 | 344693 | BioLegend | AB_2941523 |
| CD8 | BUV805 | SK1 | 612889 | BD Biosciences | AB_2833078 |
| CD11b | Spark UV 387 | ICRF44 | 301365 | BioLegend | AB_2922528 |
| CD11c | eFluor 450 | 3.9 | 48-0116-41 | Thermo Fisher | AB_11218498 |
| CD14 | Spark Blue 550 | 63D3 | 367147 | BioLegend | AB_2820021 |
| CD16 | BUV496 | 3G8 | 612944 | BD Biosciences | *AB_2870224* |
| CD19 | Spark NIR 685 | HIB19 | 302269 | BioLegend | AB_2860769 |
| CD20 | Spark Violet 538 | 2H7 | 302373 | Biolegend | AB_2941465 |
| CD24 | cFluor YG610 | SN3 | R7-20658 | Cytek Biosciences | *NA* |
| CD25 | cFluor BYG710 | 4E3 | R7-20660 | Cytek Biosciences | *NA* |
| CD27 | APC-H7 | M-T271 | 560223 | BD Biosciences | AB_1645473 |
| CD28 | BV650 | CD28.2 | 302945 | BioLegend | AB_2616854 |
| CD38 | BUV737 | HB7 | 612825 | BD Biosciences | AB_2870149 |
| CD39 | BUV661 | TU66 | 569788 | BD Biosciences | AB_3685324 |
| CD45 | PerCP | 2D1 | 368505 | BioLegend | AB_2566357 |
| CD45RA | BUV395 | 5H9 | 569489 | BD Biosciences | AB_3685102 |
| CD56 | cFluor YG584 | 5.1H11 | R7-20786 | Cytek Biosciences | *NA* |
| CD57 | cFluor B532 | HNK-1 | R7-20656 | Cytek Biosciences | *NA* |
| CD95 | PE/Cy5 | DX2 | 305610 | BioLegend | AB_314548 |
| CD103 | PE/Fire 640 | Ber-ACT8 | 350243 | BioLegend | AB_2924550 |
| CD123 | Super Bright 436 | 6H6 | 62-1239-42 | Thermo Fisher | AB_2662727 |
| CD127 | cFluor R720 | A019D5 | R7-20664 | Cytek Biosciences | *NA* |
| CD161 | APC | HP-3G10 | 17-1619-42 | Thermo Fisher | AB_10597894 |
| CD196 (CCR6) | BV711 | G034E3 | 353435 | BioLegend | AB_2629607 |
| CD195 (CCR5) | BUV563 | 2D7/CCR5 | 741401 | BD Biosciences | AB_2870893 |
| CD197 (CCR7) | BV421 | G043H7 | 353207 | BioLegend | AB_10915137 |
| CD185 (CXCR5) | BV750 | RF8B2 | 569501 | BD Biosciences | AB_3685111 |
| CD183 (CXCR3) | PE/Cy7 | G025H7 | 353719 | BioLegend | AB_11218804 |
| CD226 (DNAM-1) | Real Blue 780 | DX11 | 755549 | BD Biosciences | AB_3687902 |
| CD279 (PD-1) | BV785 | EH12.2H7 | 329929 | BioLegend | AB_11218984 |
| CD314 | BUV615 | 1D11 | 751232 | BD Biosciences | AB_2875253 |
| CD366 (TIM-3) | BB515 | 7D3 | 565569 | BD Biosciences | *AB_2744368* |
| CD337 | PE/Dazzle 594 | P30-15 | 325231 | BioLegend | AB_2814184 |
| GPR56 | PE | CG4 | 358203 | BioLegend | AB_2562083 |
| HLA-DR | BV480 | L203 | 752499 | BD Biosciences | AB_2917492 |
| IgD | cFluor BYG750 | IgD26 | R7-20662 | Cytek Biosciences | *NA* |
| IgG | BV605 | G18-145 | 563246 | BD Biosciences | AB_2738092 |
| IgM | BV570 | MHM-88 | 314517 | BioLegend | AB_10913816 |
| KLRG1 | APC/Fire 810 | SA231A2 | 367731 | BioLegend | AB_2894495 |
| TIGIT | PE/Fire 810 | A15153G | 372745 | BioLegend | AB_2924566 |
| TCRγδ | PerCP-Vio700 | REA591 | 130-114-040 | Miltenyi | AB_2733073 |
| Annexin V  conjugate | AF350 | - | A23202 | Thermo Fisher | *NA* |

**Antibody Validation.** High dimensional spectral flow cytometry was performed following a published and validated OMIP-109 45-color panel (Park et al. 2024). All antibodies were therefore used according to the established OMIP-109 protocol with minor modifications. In our panel, we added an Annexin V conjugate to the panel (Thermo Fisher, A23202), thoroughly validated by the manufacturer for specificity, sensitivity and reproducibility. Further in-house titration of the antibodies was performed to optimize staining conditions for our experiments. Instrument setup, compensation layout and gating strategies followed the original OMIP-109 protocol.

NA indicates that no corresponding RRID entry was identified in the RRID database at the time of review.

**Supplementary Table 4. Immune Cell Cluster Annotation**

| **Cell population** | **Markers used for annotation** |
| --- | --- |
| CD8+ TCM | CD3+CD8+CD45RA-CCR7+CD28+CD27+ |
| CD8+ TEMRA | CD3+CD8+CD45RA+CCR7-CD28-CD27- |
| CD8+ TEM CD28-CD27- | CD3+CD8+CD45RA-CCR7-CD28-CD27- |
| CD8+ TEM CD28+CD27+ | CD3+CD8+CD45RA-CCR7-CD28+CD27+ |
| CD8+ TN | CD3+CD8+CD45RA+CCR7+CD28+CD27+ |
| CD4+ TCM | CD3+CD4+CD45RA-CCR7+CD28+CD27+ |
| CD4+ TN | CD3+CD4+CD45RA+CCR7+CD28+CD27+ |
| CD4+ TEM CD28+CD27- | CD3+CD4+CD45RA-CCR7-CD28+CD27- |
| CD4+ TEM CD28-CD27- | CD3+CD4+CD45RA-CCR7-CD28-CD27- |
| Tregs | CD3+CD4+CD25high |
| gdTCR CD45RA+ | CD3+gdTCR+CD45RA+ |
| gdTCR CD45RA- | CD3+gdTCR+CD45RA- |
| DN T cells | CD3+CD4-CD8- |
| DN T cells 2 | CD3+CD4-CD8- |
| NKT cells | CD3+CD8+CD56+ |
| NK CD56+CD16+CD57low | CD3-CD16+CD56+CD57- |
| NK CD56+CD16+ CD57high | CD3-CD16+CD56+CD57+ |
| NK CD56+CD16- | CD3-CD56+CD16- |
| CD56dimCD16+ CD24+CXCR3+ | CD3-CD16+CD56dim CD24+CXCR3+ |
| IgG+ B cells | CD19+CD20+HLA-DR+CD24+CD38-CD39+IgG+ |
| IgM+ IgD+ CD38+ B cells | CD19+CD20+IgM+IgD+IgG-CD38+ |
| IgM+ IgD+ CD38- B cells | CD19+CD20+IgM+IgD+IgG-CD38- |
| IgM+ IgD+ CD38- B cells 2 | CD19+CD20+IgM+IgD+IgG-CD38- |
| Plasmablasts | CD19lowCD20low HLA-DR+CD27+CD38+CD39+ |
| Classical Monocytes | HLA-DR+CD11b+CD14+CD16- |
| Classical Monocytes 2 | HLA-DR+CD11b+CD14+CD16- |
| Classical Monocytes 3 | HLA-DR+CD11b+CD14+CD16- |
| Intermediate Monocytes | HLA-DR+CD11b+CD14+CD16+ |
| Intermediate Monocytes 2 | HLA-DR+CD11b+CD14+CD16+ |
| CD11c+CD16+ DCs | HLA-DR+CD11c+CD16+ |
| CD11c+CD1c+ DCs | HLA-DR+CD11c+CD1c+ |
| pDCs | HLA-DR+CD11c-CD123+ |
| DR- Basophils | HLA-DR-CD123+CD11c-CD56-CD16- |
| MAIT cells | CD3+CD8+CD27+CD28+CD161highCCR5+CCR6+ |
